# Prediction of Early Mortality Among Children With Moderate or Severe Traumatic Brain Injury Based on a Nomogram Integrating Radiological and Inflammation-Based Biomarkers

**DOI:** 10.3389/fneur.2022.865084

**Published:** 2022-05-20

**Authors:** Pingyi Zhu, Nimo Mohamed Hussein, Jing Tang, Lulu Lin, Yu Wang, Lan Li, Kun Shu, Pinfa Zou, Yikai Xia, Guanghui Bai, Zhihan Yan, Xinjian Ye

**Affiliations:** ^1^Department of Radiology, The Second Affiliated Hospital and Yuying Children's Hospital of Wenzhou Medical University, Wenzhou, China; ^2^Wenzhou Key Laboratory of Basic Science and Translational Research of Radiation Oncology, Wenzhou, China

**Keywords:** early mortality, moderate or severe traumatic brain injury, pediatrics, nomogram, radiology, inflammation

## Abstract

Inflammation-based scores have been increasingly used for prognosis prediction in neurological diseases. This study aimed to investigate the predictive value of inflammation-based scores combined with radiological characteristics in children with moderate or severe traumatic brain injury (MS-TBI). A total of 104 pediatric patients with MS-TBI were retrospectively enrolled and randomly divided into training and validation cohorts at a 7:3 ratio. Univariate and multivariate logistic regression analyses were performed to identify independent predictors of prognosis in pediatric patients with MS-TBI. A prognostic nomogram was constructed, and its predictive performance was validated in both the training and validation cohorts. Sex, admission platelet-to-lymphocyte ratio, and basal cistern status from initial CT findings were identified as independent prognostic predictors for children with MS-TBI in multivariate logistic analysis. Based on these findings, a nomogram was then developed and its concordance index values were 0.918 [95% confidence interval (CI): 0.837–0.999] in the training cohort and 0.86 (95% CI: 0.70–1.00) in the validation cohort, which significantly outperformed those of the Rotterdam, Marshall, and Helsinki CT scores. The proposed nomogram, based on routine complete blood count and initial CT scan findings, can contribute to individualized prognosis prediction and clinical decision-making in children with MS-TBI.

## Introduction

Traumatic brain injury (TBI) is a major social and clinical issue because it is one of the leading causes of mortality or permanent disability in children and adolescents worldwide (1). Pediatric TBI has exhibited various unique characteristics compared with adult TBI, which may be explained by age-related anatomical and physiological differences (2). While the clinical management of TBI has improved significantly in recent years, children with moderate or severe TBI (MS-TBI) continue to have poor clinical outcomes (2).

Predicting outcomes is critical for satisfactory pediatric TBI care. Undeniably, individual prognosis prediction contributes to improved clinical decision-making and helps minimize secondary brain injury (SBI). Computerized tomography (CT) is routinely performed to evaluate structural lesions in pediatric TBI because of its availability and speed. Indeed, it has been studied for its predictive value for mortality and functional outcomes in both individual injury characteristics and composite grading systems, including the Rotterdam CT score (3), Marshall CT classification (4), and Helsinki CT scoring system (5). However, few studies have comprehensively examined and compared the predictive values of admission CT imaging characteristics in pediatric TBI.

Hemodynamic alterations and systemic inflammation are always present in patients who sustain TBI (6). Additionally, complete blood counts are frequently performed as non-invasive laboratory procedures in clinical practice. Peripheral blood counts, including neutrophil, lymphocyte, and platelet counts, have previously been used to assess central nervous system and peripheral inflammation following injury (7). Recently, several inflammation-based prognostic scores, including the neutrophil-to-lymphocyte ratio (NLR) (8), platelet-to-lymphocyte ratio (PLR) (9), lymphocyte-to-monocyte ratio (LMR) (10) and systemic immune-inflammation index (SII) (11) have been demonstrated to accurately predict outcomes in patients with neurological diseases such as stroke, subarachnoid hemorrhage (SAH), and Alzheimer's disease (12–14). Indeed, these are low-cost and reproducible diagnostics that can be quickly calculated from a blood sample collected under simple laboratory conditions and can identify the mortality risk associated with TBI. However, no study has evaluated the predictive value of these scores in pediatric TBI.

Therefore, the purpose of this study was to retrospectively evaluate and compare the prognostic values of initial CT scan findings and blood test-based inflammation scores in a cohort of 104 children with TBI. Moreover, we established an accessible and easy-to-use nomogram to predict early mortality in pediatric TBI based on intake examinations.

## Materials and Methods

### Patients

**Figure 1:**
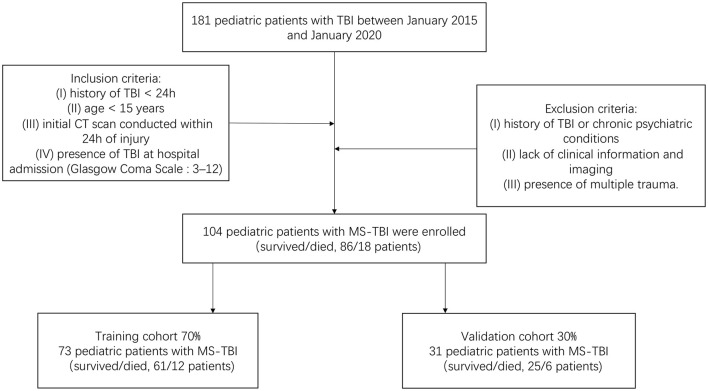
Flowchart of patient enrollment and scheme for analysis.

We retrospectively screened 104 pediatric patients with MS-TBI admitted to our hospital, the 2nd Affiliated Hospital of the Wenzhou Medical University Emergency Department, between January 2015 and January 2020. This study was approved by The Research Ethics Committee of the 2nd Affiliated Hospital of Wenzhou Medical University and complied with the standards of the Declaration of Helsinki. Informed consent was obtained from each patient for using their data for research. The inclusion criteria of our study were as follows: (I) history of TBI <24 h, (II) age <15 years, (III) initial CT scan conducted within 24 h of injury, (IV) presence of TBI at hospital admission (Glasgow Coma Scale [GCS]: 3–12). The exclusion criteria were as follows: (I) history of TBI or chronic psychiatric conditions, (II) lack of clinical information and imaging, and (III) presence of multiple trauma. All pediatric patients were assessed and treated according to standard procedures. Early mortality was defined as death at hospital discharge (15, 16). Patients were randomly divided into a training cohort (*N* = 73) and validation cohort (*N* = 31) in a 7:3 ratio. The training cohort was used to screen variables and construct the model. The validation cohort was used to validate the results obtained from the training cohort. The flowchart of patient enrollment and scheme for analysis is shown in Figure 1.

### Clinical and Radiological Variables

Clinical variables were retrospectively retrieved from the electronic medical records, including patient characteristics (age, sex, and GCS score), blood test results (neutrophil, lymphocyte, monocyte, red blood cell, white blood cell and platelet counts, hemoglobin, hematocrit (HCT), mean corpuscular volume (MCV), red cell distribution width–coefficient of variation (RDW-CV), and radiological variables (hemorrhagic mass volume, intraventricular hemorrhage (IVH), SAH, midline shift, subdural hematoma (SDH), epidural hematoma (EDH), intracerebral hematoma, hemorrhagic brain contusion, and basal cistern status). All blood samples were collected before treatment at admission. All initial CT data obtained within 24 h of injury were evaluated independently by two neuroradiologists. A consensus was used to settle disagreements. CT scans were performed using a GE Light-Speed VCT 64 slice scanner (GE Healthcare, Milwaukee, WI, USA). The CT scan parameters were a tube voltage of 120 kV, automatic tube current modulation, and a layer thickness of 5 mm.

Rotterdam scores are based on the following CT presentations: whether the basal cistern was categorized as normal, compressed, or absent; whether the midline shift was <5 mm or <5 mm; whether EDH was absent or present; and whether SAH/IVH was absent or present. Marshall CT scores use the three CT findings (midline shift, basal cisterns status, and lesion volume) and the type of hemorrhagic mass management (whether it was surgically evacuated or not). Helsinki CT scores were classified according to the level of TBI severity, as evaluated by the authors (Supplementary Table 1). In addition, the inflammation-based prognostic scores including the SII, NLR, PLR, and LMR were calculated and are described in Supplementary Table 2. The optimal threshold value for each score and blood test parameters was determined by performing an analysis of the receiver operating characteristic (ROC) for early mortality status. The value with the maximum Youden index was chosen as the optimal cut-off value.

### Statistics

Data are presented as median with interquartile range (IQR) for continuous variables and as counts with percentages for categorical variables. Univariate and multivariate logistic regression analyses were used to identify independent risk factors for early mortality in the training cohort. All variables in the univariate analysis (*P* < 0.05) were included in the multivariate analysis, and a backward stepwise selection was performed. The nomogram for predicting early mortality was developed based on variables that were considered statistically significant in the multivariate analysis. The ROC analysis and concordance index (C-index) value were applied to measure the discrimination performance of the nomogram in comparison with current CT scoring systems in both the training and validation cohorts.

All statistical analyses were performed using Stata version 15.1 (StataCorp, College Station, TX, USA) and R version 4.1.1. Differences were considered statistically significant when the two-tailed *P*-value was < 0.05.

## Results

### Patient Characteristics

The entire cohort consisted of 104 patients (60 male and 44 female) with MS-TBI. The median age of the patients was 6 (IQR: 3.5–8) years. The median GCS score was 7 (IQR: 5–9). The primary reason for trauma was fall from height (75 patients, 72.1%), followed by motor vehicle/motor cycle crashes (27 patients, 26.0%), and violence (2 patients, 1.9%).

Among these pediatric patients, the basal cistern was normal in 18 (17.3%) patients, compressed in 49 (47.1%) patients, and absent in 37 (35.6%) patients. EDH, SDH, and intracerebral hematoma were observed in 71 (68.3%), 49 (47.1%), and 71 (68.3%) patients, respectively. SAH and IVH were present in 98 (94.2%) and 17 (16.3%) patients, respectively. Moreover, the midline shift was > 5 mm in 24 patients (23.1%). Regarding the CT scoring systems, the median values of the Rotterdam, Marshall, and Helsinki CT scores were 4 (IQR: 3–5), 5 (IQR: 2–5), and 3 (IQR: 1–6), respectively. At the time of discharge from the hospital, 86 children survived, while 18 died, resulting in a 17.3% mortality rate. Other clinicopathological characteristics, including blood test variables, are summarized in Table 1.

**Table 1:** Characteristics of the patients in the training and validation cohorts.

**Variable**	**No. of patients** (**%) or Median** (**IQR)**		
	**Entire cohort (*N =* 104)**	**Training cohort (*N =* 73)**	**Validation cohort (*N =* 31)**
Age (year)	6 (3.5–8)	6 (4–8)	5 (3–7)
**Sex**
-male	60 (57.7)	45 (61.6)	15 (48.4)
-female	44 (42.3)	28 (38.4)	16 (51.6)
GCS Grade	7 (5–9)	7 (5–9)	8 (5–9)
**The basal cistern**
-normal	18 (17.3)	14 (19.2)	4 (12.9)
-compressed	49 (47.1)	34 (46.6)	15 (48.4)
-absent	37 (35.6)	25 (34.3)	12 (38.7)
**The midline shift**
- ≤ 5 mm	80 (76.9)	57 (78.1)	23 (74.2)
-> 5 mm	24 (23.1)	16 (21.9)	8 (25.8)
**EDH**
-absent	33 (31.7)	23 (31.5)	10 (32.3)
-present	71 (68.3)	50 (68.5)	21 (67.8)
**SAH**
-absent	6 (5.8)	3 (4.1)	3 (9.7)
-present	98 (94.2)	70 (95.9)	28 (90.3)
**SDH**
-absent	55 (52.9)	40 (54.8)	15 (48.4)
-present	49 (47.1)	33 (45.2)	16 (51.6)
**IVH**
-absent	87 (83.7)	61 (83.6)	26 (83.9)
-present	17 (16.3)	12 (16.4)	5 (16.1)
**Intracerebral hematoma**
-absent	33 (31.7)	24 (32.9)	9 (29.0)
-present	71 (68.3)	49 (67.1)	22 (71.0)
**Hemorrhagic mass volumes** **>25 mL**
-absent	92 (88.5)	63 (86.3)	29 (93.6)
-present	12 (11.5)	10 (13.7)	2 (6.5)
Rotterdam scores	4 (3–5)	4 (3–5)	4 (3–5)
-II	5 (4.8)	4 (5.5)	1 (3.2)
-III	27 (26.0)	19 (26.0)	8 (25.8)
-IV	38 (36.5)	29 (39.7)	9 (29.0)
-V	23 (22.1)	13 (17.8)	10 (32.3)
-VI	11 (10.6)	8 (11.0)	3 (9.7)
Marshall scores	5 (2–5)	5 (2–5)	5 (3–5)
-II	32 (30.8)	25 (34.3)	7 (22.6)
-III	10 (9.6)	8 (11.0)	2 (6.5)
-IV	5 (4.8)	3 (4.1)	2 (6.5)
-V	44 (42.4)	27 (37.0)	17 (54.8)
-VI	13 (12.5)	10 (13.7)	3 (9.7)
Helsinki CT scores	3 (1–6)	3 (1–6)	3 (1–5)
White blood cell counts (x10^9^/L)	22.2 (15.1–26.8)	19.6 (13.5–25.3)	25.4 (21–29.3)
Neutrophil counts (x10^9^/L)	16.9 (10.1–22.2)	15.0 (8.1–21.2)	19.1 (12.8–25.1)
Lymphocyte counts (x10^9^/L)	2.7 (1.3–5.0)	2.4 (1.3–4.7)	3.1 (1.5–5.6)
Monocyte counts (x10^9^/L)	1.0 (0.7–1.4)	1.0 (0.6–1.4)	1.2 (0.8–1.5)
Hb (g/L)	106 (92–121)	109 (93–122)	99 (86–120)
Red blood cell counts (x10^12^/L)	3.9 (3.3–4.3)	4.0 (3.4–4.3)	3.7 (3.1–4.2)
HCT	0.32 (0.27–0.36)	0.32 (0.27–0.36)	0.30 (0.26–0.35)
MCV (fl)	82.9 (79.6–85.5)	82.5 (79.7–85.2)	83.3 (79.5–86.2)
RDW-CV (%)	12.9 (12.4–13.4)	13.0 (12.5–13.7)	12.7 (12.4–13.3)
Platelet counts	289.5 (211.5–249.5)	280.0 (210.0–335.0)	310.0 (217.0–368.0)
SII	1513.2 (665.7–3405.1)	1280.4 (614.9– 3163.0)	1991.6 (820.9–3517.6)
NLR	5.1 (2.4–11.6)	4.5 (2.4–11.5)	6.4 (3.0–13.6)
PLR	96.7 (55.8–190.5)	97.6 (58.3–194.0)	88.3 (46.5–172.1)
LMR	2.6 (1.5–5.7)	2.6 (1.5–6.2)	2.4 (1.4–5.3)

*IQR, interquartile range; GCS, Glasgow Coma Scale; EDH, epidural hematoma; SAH, subarachnoid hemorrhage; SDH, subdural hematoma; IVH, intraventricular hemorrhage; Hb, hemoglobin; HCT, Hematocrit; MCV, mean corpuscular volume; RDW-CV, red cell distribution width–coefficient of variation; SII, systemic immune-inflammation index; NLR, neutrophil to lymphocyte ratio; PLR, platelet to lymphocyte ratio; LMR, lymphocyte to monocyte ratio*.

### Risk Factors for Early Mortality in Children With MS-TBI

The optimal cut-off values for blood test parameters were determined by ROC analysis and are presented in Table 2. In the training cohort, our univariate analysis revealed that basal cistern (OR = 6.84, 95% confidence interval [CI]: 1.83–25.59, *P* = 0.004), sex (OR = 6.15, 95% CI: 1.50–25.23, *P* = 0.012), MCV (OR = 0.15, 95% CI: 0.04–0.62, *P* = 0.009), HCT (OR = 0.16, 95% CI: 0.04–0.62, *P* = 0.008), RDW-CV (OR = 3.69, 95% CI: 1.02–13.37, *P* = 0.047), NLR (OR = 3.62, 95% CI: 1.01–12.99, *P* = 0.048), PLR (OR = 9.17, 95% CI: 2.24–37.58, *P* = 0.002) and LMR (OR = 0.18, 95% CI: 0.04–0.90, *P* = 0.036) were associated with early mortality in children with MS-TBI. Furthermore, multivariate analysis demonstrated that basal cistern (OR = 22.12, 95% CI: 2.43–201.58, *P* = 0.006), sex (OR = 8.38, 95% CI: 1.30–54.18, *P* = 0.026), and PLR (OR = 63.22, 95% CI: 4.50–888.79, *P* = 0.002) were independent risk factors for early mortality in this population (Table 3).

**Table 2:** Receiver operating characteristic curve analyses of the blood test-based parameters.

**Variables**	**AUC**	**Cut-off value**	***P*-value**
White blood cell counts (x10^9^/L)	0.60 (0.45–0.75)	20	0.588
Hb (g/L)	0.71 (0.61–0.81)	103	0.126
Red blood cell counts (x10^12^/L)	0.64 (0.50–0.78)	3.89	0.701
HCT	0.74 (0.63–0.86)	0.293	0.061
RDW-CV (%)	0.58 (0.42–0.75)	13.4	0.047
MCV (fl)	0.68 (0.55–0.80)	81.3	0.068
SII	0.71 (0.58–0.84)	1,722	0.106
NLR	0.64 (0.50–0.79)	3.35	0.202
PLR	0.73 (0.58–0.88)	49.56	0.204
LMR	0.69 (0.54–0.83)	2.375	0.020

*AUC, area under the curve; Hb, hemoglobin; HCT, Hematocrit; RDW-CV, red cell distribution width–coefficient of variation; MCV, mean corpuscular volume; SII, systemic immune-inflammation index; NLR, neutrophil to lymphocyte ratio; PLR, platelet to lymphocyte ratio; LMR, lymphocyte to monocyte ratio*.

**Table 3:** Univariate and multivariate analysis of early pediatric mortality in the training cohort.

	**Univariate analysis**		**Multivariate analysis**	
	**OR (95% CI)**	***P*-value**	**OR (95% CI)**	***P*-value**
Age	1.00 (0.95–1.05)	0.940		
Sex	6.15 (1.50–25.23)	0.012	8.38 (1.30–54.18)	0.026
Basal cistern	6.84 (1.83–25.59)	0.004	22.12 (2.43–201.58)	0.006
Midline shift	0.67 (0.13–3.43)	0.632		
EDH	2.63 (0.53–13.10)	0.239		
SDH	0.90 (0.26–3.15)	0.868		
IVH	1.93 (0.44–8.51)	0.387		
Intracerebral hematoma	2.82 (0.57–14.05)	0.206		
Hemorrhagic mass volumes greater than 25 mL	1.54 (0.28–8.53)	0.619		
White blood cell counts	2.36 (0.64–8.66)	0.197		
Hb	0.32 (0.09–1.20)	0.091		
Red blood cell counts	0.53 (0.15–1.86)	0.322		
HCT	0.16 (0.04–0.62)	0.008		
MCV	0.15 (0.04–0.62)	0.009		
RDW-CV	3.69 (1.02–13.37)	0.047		
SII	0.21 (0.04–1.02)	0.053		
NLR	3.62 (1.01–12.99)	0.048		
PLR	9.17 (2.24–37.58)	0.002	63.22 (4.50–888.79)	0.002
LMR	0.18 (0.04–0.90)	0.036		

*OR, odds ratio; CI, confidence interval; EDH, epidural hematoma; SAH, subarachnoid hemorrhage; SDH, subdural hematoma; IVH, intraventricular hemorrhage; Hb, hemoglobin; HCT, Hematocrit; MCV, mean corpuscular volume; RDW-CV, red cell distribution width–coefficient of variation; SII, systemic immune-inflammation index; NLR, neutrophil to lymphocyte ratio; PLR, platelet to lymphocyte ratio; LMR, lymphocyte to monocyte ratio*.

### Development and Validation of the Nomogram

**Figure 2:**
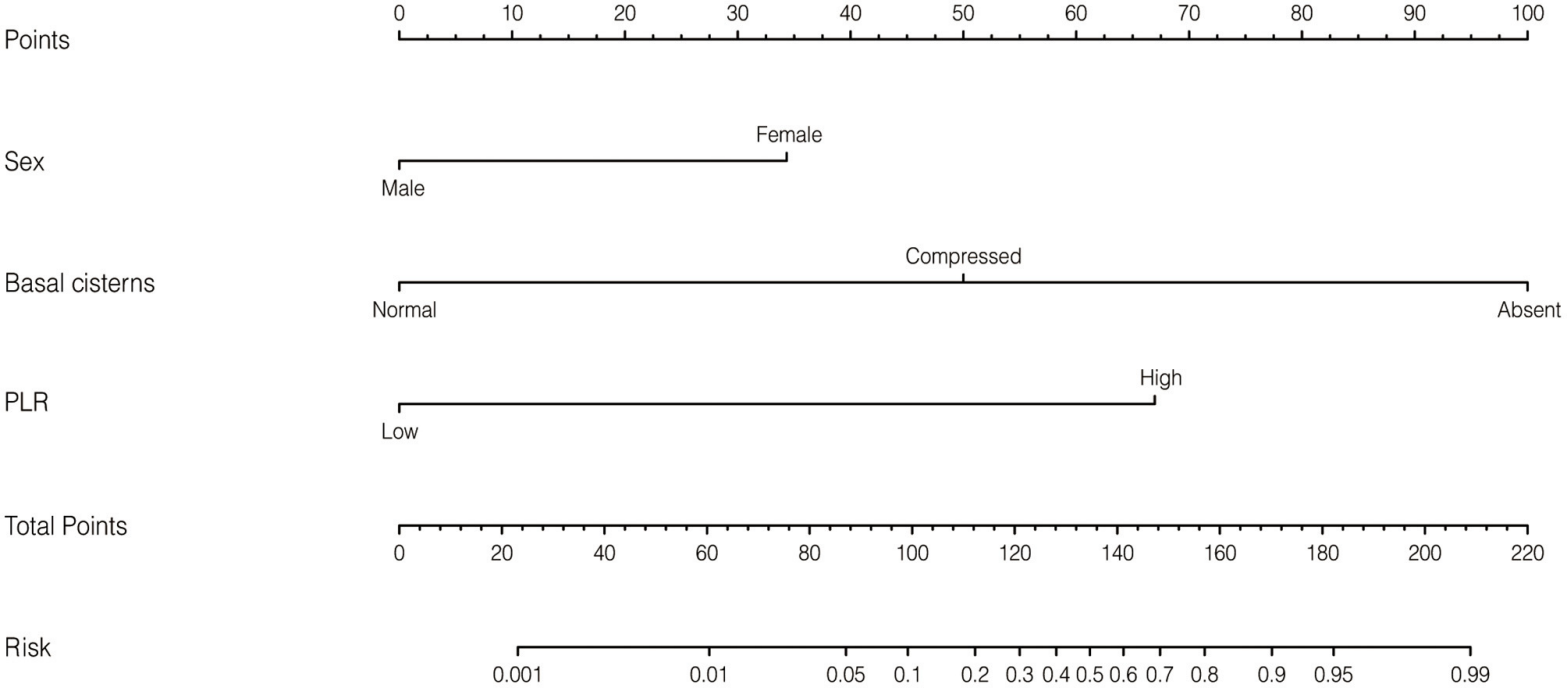
Nomogram to predict early mortality in pediatric patients with moderate or severe traumatic brain injury (MS-TBI).

**Figure 3:**
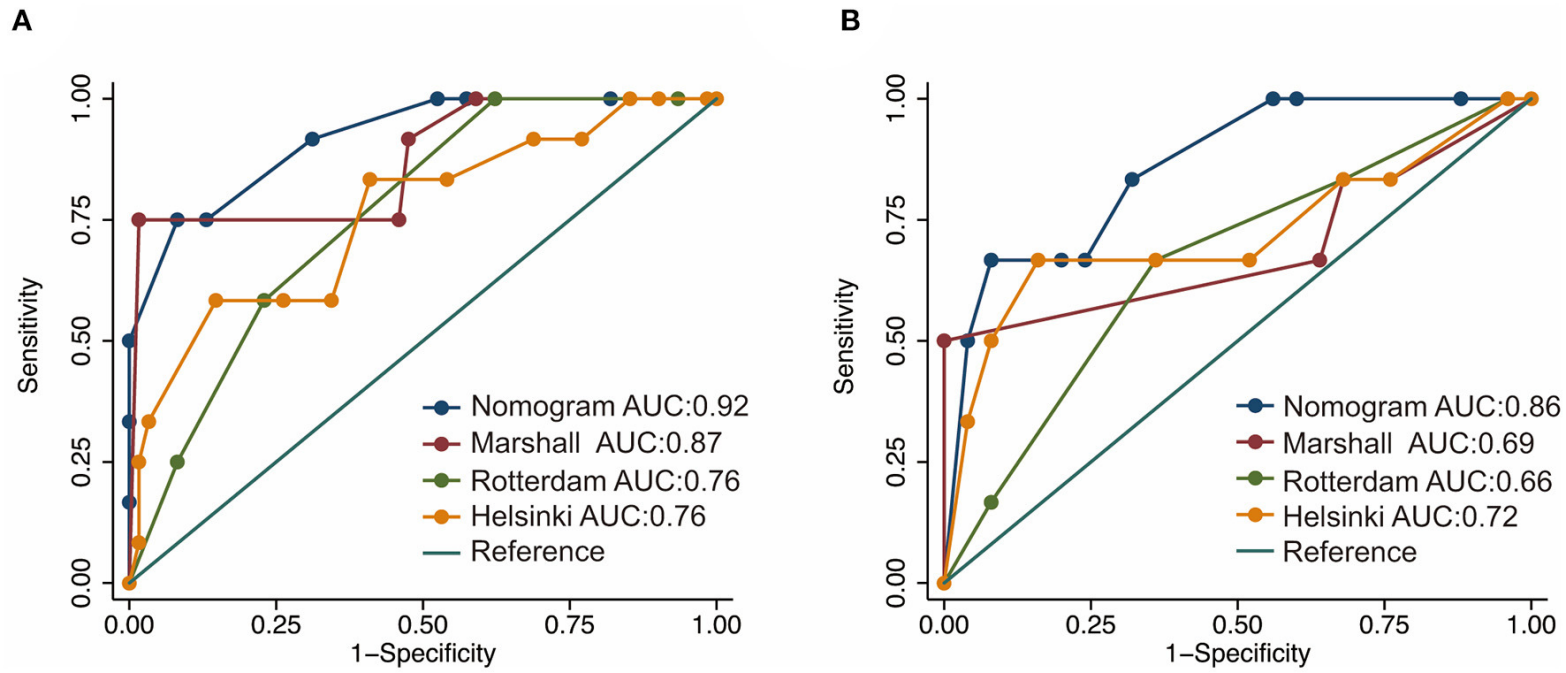
Receiver operating characteristic (ROC) curves of the nomogram and current CT scoring systems in the training **(A)** and validation cohorts **(B)**.

Based on the independent risk factors identified by the multivariate logistic regression analysis in the training cohort, we constructed a nomogram to predict the risk of early mortality in pediatric patients with MS-TBI. Each variable was assigned a score based on its β coefficient (Figure 2). The nomogram had C-index values of 0.918 in the training cohort and 0.86 in the validation cohort (Table 4). The area under the curve (AUC) values demonstrated the excellent predictive ability of our current nomogram [training cohort: AUC = 0.92 (95% CI: 0.84–1.00); validation cohort: AUC = 0.86 (95% CI: 0.70–1.00)], which significantly outperformed the currently used Rotterdam, Marshall, and Helsinki CT scores (Table 4 and Figure 3).

**Table 4:** Performance of the nomogram in predicting early mortality in pediatric patients with TBI comparing with current CT scoring systems.

**Performance parameter**	**Training cohort**				**Validation cohort**			
	**Nomogram**	**Marshall**	**Rotterdam**	**Helsinki**	**Nomogram**	**Marshall**	**Rotterdam**	**Helsinki**
AUC	0.92	0.87	0.76	0.76	0.86	0.69	0.66	0.72
95% CI low	0.84	0.74	0.64	0.60	0.70	0.38	0.41	0.42
95% CI high	1.00	1.00	0.88	0.92	1.00	1.00	0.91	1.00
C-index	0.92	0.87	0.76	0.76	0.86	0.69	0.66	0.72

*TBI, traumatic brain injury; CT, computerized tomography; AUC, area under the curve; CI, confidence interval*.

### Optimal Cut-Off Value of the Nomogram

To facilitate the use of the newly developed nomogram, we determined the optimal threshold value of the nomogram to be 135 points based on the ROC analysis of the entire cohort. Patients were divided into low-risk (score ≤ 135 points) and high-risk (score >135 points) groups. In the training cohort, the early mortalities were 5.1% in the low-risk group and 64.3% in the high-risk group. In the validation cohort, the early mortalities were 9.1% in the low-risk group and 44.4% in the high-risk group. Therefore, the current nomogram could serve as an easy-to-use model with good performance for risk discrimination in pediatric patients with MS-TBI.

## Discussion

TBI is an injury to the cranium and its intracranial components produced by an external mechanical force, which changes the brain's structure and function (17). Primary and SBI is a pathological process following TBI (18). Primary brain injury occurs when the brain tissue is subjected to mechanical force, resulting in axonal, vascular, and glial cell damage. SBI occurs due to the initiation of inflammatory cascades by the release of different inflammatory chemicals and neurotransmitters from the injured neuronal and glial cells in the brain (19).

Currently, the GCS is the most commonly used technique for assessing the severity of TBI. On the other hand, GCS is a collection of clinical descriptive data that does not include structural information about cerebral abnormalities. Additionally, the GCS had a low prognostic value in the early stages post-injury, and neurological examination in children is challenging. More importantly, a history of neurological illnesses and the presence of an EDH during the intermediate waking state may affect the accuracy of GCS (20). As a result, finding efficient biomarkers for the prognosis of pediatric TBI from admission examinations is critical. The predictive value of inflammation scores in pediatric TBI cases with a combination of intake CT findings is still unclear and has not been extensively explored. To bridge this gap, the present study, for the first time, compared the predictive values of various inflammation prognostic scores and CT scan parameters in a large cohort of 104 children with MS-TBI.

In this study, ROC analysis was applied to determine the optimal cut-off values of various blood test parameters (Table 2). In the training cohort, the univariate and multivariate logistic regression analyses demonstrated that the basal cistern, sex, and PLR could independently predict early death in pediatric patients with MS-TBI (Table 3). To further facilitate clinical practice, we constructed a nomogram for prognosis prediction (Figure 2). The C-index and AUCs demonstrated that the nomogram significantly outperformed current CT scoring systems, including the Rotterdam CT, Marshall CT, and Helsinki CT scores in both the training and validation cohorts (Table 4). All these data demonstrated that the current nomogram could serve as a preferable model for pediatric patients with MS-TBI.

The mechanism underlying the excellent predictive value of the newly developed nomogram may be explained as follows. PLR was initially introduced to represent systemic inflammation and to estimate the prognosis of patients with cancer and autoimmune diseases (21, 22). Recent research has demonstrated that PLR is a simple parameter that can be used to predict clinical outcomes in patients with stroke, cerebral hemorrhage, and SAH (13, 23–26). In response to physiological stress, such as traumatic injury, the body produces more cortisol, whose elevated levels can result in lymphopenia (27); the higher the physiological stress, the higher the cortisol level, which results in a decrease in the body's lymphocyte count. Contrastingly, an increased lymphocyte count indicates a more targeted immune response and a more persistent inflammatory pathway (28). Moreover, lymphocytes represent a more tightly controlled inflammatory route, and apoptosis induced by lymphocytes is less harmful to neighboring cells than other cell death models caused by uncontrolled inflammation (29). Thus, a decreased lymphocyte count post-TBI may reflect the damaging effect of the inflammatory response, resulting in an exacerbated SBI post-TBI. On the other hand, along with their well-known hemostatic functions, platelets play an active role in inflammation regulation. While platelets bind to coagulation factors, they also include a large variety of inflammatory factors, such as TNF-α, interleukins, and serotonin, all of which are involved in tissue injury and repair (7). Therefore, it is reasonable to conclude that a high PLR may be associated with an increased risk of death in pediatric patients with MS-TBI.

Additionally, previous research has demonstrated that obliterated cisterns are associated with increased intracranial pressure and poor outcomes (30, 31), suggesting that the status of the cisterns can be used as an early non-invasive method for identifying patients at high risk of death or severe disability, despite the initial neurological examination indicating otherwise. Further, some studies that focused on adults explored sex differences in outcomes following moderate/severe TBIs, mainly showing an absence of differences or better outcomes in women compared to men. However, following mild TBI, most studies indicate worse psychological and global functioning outcomes in women. Biological differences, particularly sex steroids, represent another pathway that can mediate these sex differences in outcomes. Animal models suggest that the hypothalamic-pituitary-adrenal axis, which modulates the stress response, and microglia, which influence cerebral inflammation, have sex-specific responses. Indeed, these preclinical models have shown that female rodents present a stronger inflammatory response than males after TBI (32–37).

Nevertheless, this study has several limitations that are worth mentioning. First, as a single-center retrospective study, potential selection bias was inevitable, although we had performed strict inclusion criteria. Second, the cut-off values for inflammation-based indices used in this study were determined by ROC analysis based on outcome data, and further validation studies determining optimal threshold values for these inflammation indices are needed. Third, peripheral platelet and lymphocyte counts may be affected by other pathological conditions, including metabolic syndrome and renal dysfunction. Hence, the clinical significance of PLR should be cautiously explained with full consideration of any concurrent diseases.

## Conclusions

We identified sex, admission PLR, and basal cistern status from initial CT findings as independent prognostic predictors in pediatric patients with MS-TBI, which may contribute to individualized prognosis prediction and better clinical decision making. However, a larger data set from multiple centers is still needed to confirm the findings of this research and promote its clinical implementation.

## Data Availability Statement

The raw data supporting the conclusions of this article will be made available by the authors, without undue reservation.

## Ethics Statement

The studies involving human participants were reviewed and approved by the Research Ethics Committee of The Second Affiliated Hospital of Wenzhou Medical University. Written informed consent to participate in this study was provided by the participants' legal guardian/next of kin.

## Author Contributions

PZh, NH, ZY, and XY contributed to the conception of the study. LLi, JT, YX, YW, PZo, and KS performed the data collection. PZh, NH, and GB performed the data analyses and wrote the manuscript. JT, LLin, LLi, ZY, and XY helped perform the analysis with constructive discussions. All authors contributed to the article and approved the submitted version.

## Funding

This work was supported by grants from the Natural Science Foundation of Zhejiang Province (No. LY19H180003) and the National Natural Science Foundation of China (No. 82071902).

## Conflict of Interest

The authors declare thatthe research was conducted in the absence of any commercial or financial relationships that could be construed as a potential conflict of interest.

## Publisher's Note

All claims expressed in this article are solely those of the authors and do not necessarily represent those of their affiliated organizations, or those of the publisher, the editors and the reviewers. Any product that may be evaluated in this article, or claim that may be made by its manufacturer, is not guaranteed or endorsed by the publisher.

## Abbreviations

MS-TBI: moderate or severe traumatic brain injury
PLR: platelet to lymphocyte ratio
C-index: concordance index
CI: confidence interval
CT: computerized tomography
SBI: secondary brain damage progression
CNS: central nervous system
NLR: neutrophil to lymphocyte ratio
LMR: lymphocyte to monocyte ratio
SII: systemic immune-inflammation index
GCS: Glasgow Coma Scale
Hb: hemoglobin
HCT: Hematocrit
MCV: mean corpuscular volume
RDW-CV: red cell distribution width–coefficient of variation
IVH: intraventricular hemorrhage
SAH: subarachnoid hemorrhage
SDH: subdural hematoma
EDH: epidural hematoma
ROC: receiver operating characteristic
IQR: interquartile range
OR: odds ratio
AUC: area under the curve
HPA: hypothalamic–pituitary–adrenal axis.
