# Supplementary material for: Prediction of Early Mortality Among Children With Moderate or Severe Traumatic Brain Injury Based on a Nomogram Integrating Radiological and Inflammation-Based Biomarkers
Source: Front Neurol. 2022 May 20;13:865084. doi: 10.3389/fneur.2022.865084 (PMC9163313; doi:10.3389/fneur.2022.865084)
Supplement: Supplementary file 1 [file Table_1.DOCX]

**Supplementary Table 1** The CT scoring systems.

| System | Classification or component | Description |
| --- | --- | --- |
| Rotterdam CT score | Basal cisterns | 0: normal, 1: compressed, 2: absent |
|  | Midline shift | 0: no shift or ≤ 5 mm, 1: shift > 5 mm |
|  | Epidural mass lesion | 0: present, 1: absent |
|  | IVH/SAH | 0: absent, 1: present |
|  | Score | sum+1(1-6) |
| Marshall CT score | I | No visible intracranial pathology on computed tomography |
|  | II | Midline shift of 0 to 5 mm, basal cisterns remain visible, no high- or mixed-density lesions > 25 ml includes bone fragments or foreign bodies |
|  | III | Midline shift of 0 to 5 mm, basal cisterns compressed or absent, no high- or mixed-density lesions > 25 ml |
|  | IV | Midline shift > 5 mm, no high- or mixed-density lesions > 25 ml |
|  | V | any lesion surgically evacuated |
|  | VI | High- or mixed-density lesions > 25 ml, not surgically evacuated |
| Helsinki CT score | Mass lesion type, if present | Subdural hematoma: 2, intracerebral hematoma: 2, epidural hematoma: −3 |
|  | Mass lesion size | Hematoma volume > 25 cm^3^: 2 |
|  | IVH | Present: 3 |
|  | Basal cisterns | Normal: 0, compressed: 1, absent: 5 |
|  | Score | Sum (range: −3 to 14) |

**Note:** CT, computerized tomography; IVH, intraventricular hemorrhage; SAH, subarachnoid hemorrhage.

**Supplementary Table 2** Inflammation-based prognostic scores.

| Criteria |
| --- |
| Systemic immune-inflammation index (SII)  - Platelet count (×10^9^/L) × neutrophil count (×10^9^/L)/lymphocyte count (×10^9^/L) |
| Neutrophil to lymphocyte ratio (NLR)  -Neutrophil count (×10^9^/L)/lymphocyte count (×10^9^/L) |
| Platelet to lymphocyte ratio (PLR)  -Platelet count (×10^9^/L)/lymphocyte count (×10^9^/L) |
| Lymphocyte to monocyte ratio (LMR)  -Lymphocyte count (×10^9^/L)/monocyte count (×10^9^/L) |
